# Real-world comparative effectiveness of SARS-CoV-2 primary vaccination campaigns against SARS-CoV-2 infections: a federated observational study emulating a target trial in three nations

**DOI:** 10.1093/eurpub/ckaf247

**Published:** 2026-01-07

**Authors:** Marjan Meurisse, Francisco Estupiñán-Romero, Markus Perola, Teemu Paajanen, Javier González-Galindo, Nina Van Goethem, Enrique Bernal-Delgado

**Affiliations:** Department of Epidemiology and Public Health, Sciensano, Brussels, Belgium; Institut de Recherche Expérimentale et Clinique (IREC)—EPID, Université Catholique de Louvain, Brussels, Belgium; Data Science for Health Services and Policy, Instituto Aragonés de Ciencias de la Salud (IACS), Zaragoza, Spain; Department of Public Health and Welfare, Finnish Institute for Health and Welfare (THL), Helsinki, Finland; Department of Public Health and Welfare, Finnish Institute for Health and Welfare (THL), Helsinki, Finland; Data Science for Health Services and Policy, Instituto Aragonés de Ciencias de la Salud (IACS), Zaragoza, Spain; Department of Epidemiology and Public Health, Sciensano, Brussels, Belgium; Data Science for Health Services and Policy, Instituto Aragonés de Ciencias de la Salud (IACS), Zaragoza, Spain

## Abstract

To assess the impact of large-scale severe acute respiratory syndrome coronavirus 2 (SARS-CoV-2) vaccination campaigns in real-world settings across regions, we performed a reproducible cross-border comparison of the real-world effectiveness of primary vaccination in preventing SARS-CoV-2 infections across three sites: Aragon (Spain), Brussels and Wallonia (Belgium), and Finland. This observational study emulated a target trial by daily matching primary vaccinated individuals 1:1 to un- or partially vaccinated individuals using propensity scores estimated on a set of relevant confounders from January to September 2021. Matched individuals were followed up until a SARS-CoV-2 infection was contracted or a censoring event occurred. Vaccine effectiveness in preventing infections was estimated by the difference in restricted mean survival time (RMST). Primary vaccination extended the average free-of-infection time by 35.9 [95% confidence interval (CI) (34.9–37.0)], 59.6 [95% CI (59.3–60.0)], and 1.6 [95% CI (1.1–2.0)] days over 365 days in the population cohort of Aragon (Spain), Brussels and Wallonia (Belgium), and Finland, respectively. This federated population-based observational study showed the effectiveness of the SARS-CoV-2 primary vaccination campaign in prolonging the mean time to infection in the Aragon (Spain) and Brussels and Wallonia (Belgium) population cohorts. Only a minor difference over this time frame was found in Finland’s population cohort.

## Introduction

Since its identification in January 2020, the severe acute respiratory syndrome coronavirus 2 (SARS-CoV-2), causing coronavirus disease 2019 (COVID-19), has resulted in a global public health crisis. Less than 1 year after the official identification of the virus, the first SARS-CoV-2 vaccine, i.e. the BNT162b2 vaccine (Comirnaty, Pfizer–BioNTech), was scientifically evaluated and authorized in the European Union (EU) by the European Medicines Agency (EMA), shortly followed by the authorization of the mRNA-1273 (Spikevax, Moderna), ChAdOx1-S (Vaxzevria, Oxford–AstraZeneca), and Ad26.COV2.S (COVID-19 vaccine Janssen, Johnson & Johnson) vaccines [1]. At the end of 2020, following the authorization of the first vaccines, large-scale SARS-CoV-2 vaccination campaigns were initiated across Europe, resulting in high coverage of primary vaccination across the EU [2]. For example, in Spain and Finland, the first dose was administered on the 27th of December 2020, and Belgium started its vaccination campaign on 28 December 2020.

Post-authorization vaccine effectiveness (VE) studies have been conducted reusing real-world data from real-world settings in many European countries showing that having received one or two vaccine doses protects against (symptomatic) infection [3–7]. Comparative analyses of these single-centre studies is challenging given that they generally focus on a subpopulation with specific characteristics (e.g. within one country or region), use different methodologies or exposure/outcome measures, or consider different confounding factors during the analysis. More recently, a Vaccine Effectiveness, Burden and Impact Studies (VEBIS) study by Fontán-Vela *et al.* (2024) [8] did offer a cross-country assessment of real-world VE in preventing COVID-19 hospitalization following a common protocol.

The most appropriate approach to inform policy-making based on causal estimates inferred from real-world observational data is to emulate a target trial, which represents the hypothetical randomized clinical trial that could have been conducted to answer the research question [9]. The current study extends existing research by (i) emulating a target trial, (ii) using virtually all the target population (allowing us to evaluate the vaccination campaign), and (iii) performing a cross-border comparison using a reproducible methodology. In this work, conducted as part of the Horizon Europe-funded BeYond-COVID (BY-COVID) project [10], we sought to determine the real-world effectiveness of primary vaccination campaigns in preventing SARS-CoV-2 infections by leveraging data from existing COVID-19 real-world data sources from Aragon (Spain), Brussels and Wallonia (Belgium) and Finland, and adopting a federated analysis methodology [11].

## Methods

### Study design

Starting from a population cohort including all eligible population from three sites, namely Aragon (Spain), Brussels and Wallonia (Belgium), and Finland, we designed an observational study emulating a target trial to assess real-world effectiveness of SARS-CoV-2 primary vaccination campaigns in preventing SARS-CoV-2 infection during the pandemic—from the start of the primary vaccination campaigns (1 January 2021), until a year after the start of the booster campaign (1 September 2022). The methodological framework using federated causal inference with real-world data has been described elsewhere [11], and the specification of the target trial is explicitly described in Supplementary Figs S1 and S2 and Supplementary Table S1. The corresponding causal model constructed using a directed acyclic graph (DAG) is presented in Supplementary Fig. S3, and the subsequent translation into data requirements in the form of a common data model (CDM) can be consulted elsewhere [12]. The analysis code and execution instructions of the reproducible analysis pipeline is provided within a GitHub repository [13] (see documentation provided within the repository for more information [14]).

### Study participants

The study population included all individuals eligible to be vaccinated (5–115 years old) during the period of study. Individuals without any vaccination or infection records during this period were excluded, as were those with an infection before primary vaccination or before 1 January 2021. In addition, individuals non-compliant with predefined validation rules, as for example, checking ages within inclusion criteria, misreported dates, or incomplete information (see documentation provided within the GitHub repository [14]), based on the specified eligibility criteria (see Supplementary Table S1) and data requirements detailed in the CDM [12], were excluded from the study population. The data extraction periods in the respective sites are presented in Supplementary Table S2.

### Data sources

Individual-level COVID-19 cohort data and vaccination data were linked within each site using pseudonymized identifiers to create site-specific cohorts. Patient administrative information (e.g. data from insurance registries or health system user databases), comorbidities, and mortality data were additionally linked, as per the CDM requirements [12]. Authorized researchers accessed pseudonymized data in secured environments on 11 April 2023 (Aragon), 26 March 2024 (Brussels and Wallonia), and 19 April 2024 (Finland). Data sources and linkage procedures are detailed in the data management plan [15] and study protocol [16].

Ethical approval was granted for data linking in Aragon and Brussels and Wallonia. THL did not require ethical approval for processing registry data. Informed consent was waived per GDPR, based on public interest (Art. 6) and public health (Art. 9 § 2).

### Exposure groups and assignment

Within each site-cohort, daily iterative matching of exposed (completed primary vaccination regardless of the schedule) to unexposed (non/partial vaccination) individuals was performed. Matching used a ‘minimal sufficient adjustment set’: age, gender, residence area, country, foreign, essential worker, institutionalized, individual-level socio-economic status (SES), presence of comorbidities, immune status, and pregnancy identified based on the constructed causal model (see Supplementary Fig. S3). According to a prespecified decision algorithm (see Supplementary Fig. S4), missing values were imputed with predictive mean matching using the ‘mice’ R package, and list wise deletion was performed or variables excluded for the matching if data was not available: individual-level SES (Aragon), foreign and pregnancy (Brussels and Wallonia), and institutionalized, foreign and pregnancy (Finland). Matching was exact when possible; otherwise, a greedy nearest neighbour propensity-score matching was used using the ‘MatchIt’ R package (see Supplementary Fig. S5). Covariate balance was assessed using standardized mean differences (SMD).

### Follow-up period and main endpoint

The outcome was the register of a documented SARS-CoV-2 infection. Follow-up continued until infection, death, death of a matched individual, or end of the study period for all individuals included in the cohort. In addition, follow-up ended upon primary vaccination completion or booster dose of the matched primary vaccinated individual in the individuals considered as controls; and upon primary vaccination completion of the matched unexposed comparator or administration of the booster dose in individuals considered exposed (see timeline in Supplementary Fig. S6).

### Statistical analyses

Baseline characteristics of matched participants were reported. Time-to-event analysis was performed for each site, and cumulative incidence (CInc) curves were generated. Restricted mean survival time difference (RMSTD), an estimation of the effect based on the difference of the areas under the survival curve for exposed and non-exposed, was calculated over 1 year. RMSTD is an estimation of the average treatment effect (ATE), as proportional hazards assumption was not met for Cox survival models [17]. RMSTD were compared across participant regions. RMSTD was estimated using the ‘survRM2’ R package. All analyses were conducted using R programming language (see packages references in Supplementary materials) [18].

### Sensitivity analyses

E-values, calculated using the R package ‘EValue’, assessed the robustness of results to unmeasured confounding [19]. Additional sensitivity analyses in Brussels and Wallonia compared the unadjusted RMSTD estimate (matching without individual-level SES) with the adjusted RMSTD estimate (matching with individual-level SES) to explore potential residual confounding.

## Results

### Study population characteristics

**Figure 1.**
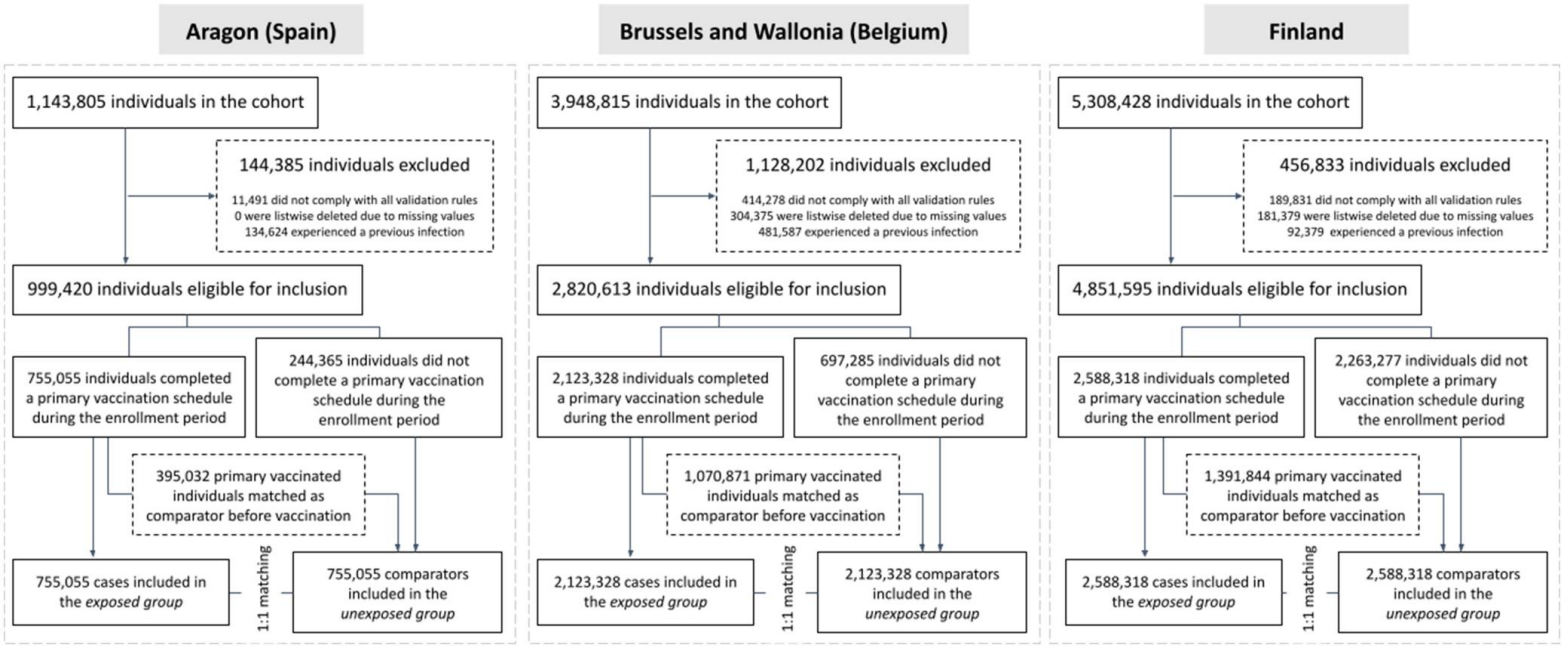
A flow diagram representing the selection process of obtaining the matched study population considered for time-to-event analysis at the different sites: Aragon (Spain), Brussels and Wallonia (Belgium), and Finland. Enrolment period: 1 January 2021, to 1 September 2021. Each day of the enrolment period, newly primary vaccinated individuals (exposed) were 1:1 matched to un- or partially vaccinated individuals (unexposed) based on a set of confounders.

Initially, the population cohorts included 1 143 805 (Aragon, Spain), 3 948 815 (Brussels and Wallonia, Belgium), and 5 308 428 (Finland) individuals (see Supplementary Table S3 for population characteristics pre-matching). After 1:1 matching, 755 055, 2 123 328, and 2 588 318 matched pairs were obtained, respectively. Selection procedures are presented in Fig. 1. HTML reports with local outputs are accessible elsewhere [20].

Table 1 shows the characteristics of the matched study population. As shown, post-matching, baseline characteristics were comparable between exposed and control groups.

**Table 1.** Characteristics of the matched study population by site [Aragon (Spain), Brussels and Wallonia (Belgium), and Finland)] and by exposure group^a^.

	Aragon (Spain)		Brussels and Wallonia (Belgium)		Finland	
	Exposed group (*N = *755 055)	Unexposed group (*N = *755 055)	Exposed group (*N = *2 123 328)	Unexposed group (*N = *2 123 328)	Exposed group (*N = *2 588 318)	Unexposed group (*N = *2 588 318)
Sex, *n* (%)						
1 (male)	360 616 (47.8%)	360 813 (47.8%)	1 019 290 (48.0%)	1 019 188 (48.0%)	1 209 685 (46.7%)	1 209 663 (46.7%)
2 (female)	394 439 (52.2%)	394 242 (52.2%)	1 104 038 (52.0%)	1 104 140 (52.0%)	1 378 633 (53.3%)	1 378 655 (53.3%)
Age (category), *n* (%)						
2 (5–9 yo)	0 (0%)	0 (0%)	35 (0.0%)	34 (0.0%)	0 (0.0%)	1 (0.0%)
3 (10–14 yo)	318 (0.0%)	318 (0.0%)	10 926 (0.5%)	10 895 (0.5%)	2242 (0.1%)	2242 (0.1%)
4 (15–19 yo)	16 314 (2.2%)	16 313 (2.2%)	81 026 (3.8%)	80 966 (3.8%)	37 872 (1.5%)	37 869 (1.5%)
5 (20–24 yo)	20 863 (2.8%)	20 857 (2.8%)	118 530 (5.6%)	118 468 (5.6%)	55 185 (2.1%)	55 181 (2.1%)
6 (25–29 yo)	24 610 (3.3%)	24 609 (3.3%)	120 852 (5.7%)	120 777 (5.7%)	79 327 (3.1%)	79 328 (3.1%)
7 (30–34 yo)	36 643 (4.9%)	36 628 (4.9%)	135 461 (6.4%)	135 357 (6.4%)	99 915 (3.9%)	99 910 (3.9%)
8 (35–39 yo)	50 711 (6.7%)	50 703 (6.7%)	141 127 (6.6%)	141 099 (6.6%)	140 313 (5.4%)	140 320 (5.4%)
9 (40–44 yo)	72 485 (9.6%)	72 461 (9.6%)	153 492 (7.2%)	153 417 (7.2%)	184 719 (7.1%)	184 717 (7.1%)
10 (45–49 yo)	79 881 (10.6%)	79 920 (10.6%)	172 450 (8.1%)	172 491 (8.1%)	195 696 (7.6%)	195 716 (7.6%)
11 (50–54 yo)	76 979 (10.2%)	77 022 (10.2%)	187 065 (8.8%)	187 339 (8.8%)	246 049 (9.5%)	246 075 (9.5%)
12 (55–59 yo)	74 495 (9.9%)	74 525 (9.9%)	195 884 (9.2%)	196 027 (9.2%)	270 896 (10.5%)	270 930 (10.5%)
13 (60–64 yo)	66 025 (8.7%)	66 019 (8.7%)	195 720 (9.2%)	195 937 (9.2%)	280 746 (10.8%)	280 777 (10.8%)
14 (65–69 yo)	5885 (7.5%)	56 873 (7.5%)	181 337 (8.5%)	181 386 (8.5%)	296 567 (11.5%)	296 490 (11.5%)
15 (70–74 yo)	53 710 (7.1%)	53 708 (7.1%)	159 808 (7.5%)	159 781 (7.5%)	300 864 (11.6%)	300 835 (11.6%)
16 (75–79 yo)	45 802 (6.1%)	45 795 (6.1%)	111 346 (5.2%)	111 268 (5.2%)	179 299 (6.9%)	179 299 (6.9%)
17 (80–84 yo)	33 447 (4.4%)	33 428 (4.4%)	76 969 (3.6%)	76 862 (3.6%)	125 315 (4.8%)	125 315 (4.8%)
18 (≥85 yo)	45 887 (6.1%)	45 876 (6.1%)	81 300 (3.8%)	81 224 (3.8%)	93 313 (3.6%)	93 313 (3.6%)
Pregnancy, *n* (%)						
Yes	13 395 (1.8%)	13 337 (1.8%)	–	–	–	–
No	741 660 (98.2%)	741 718 (98.2%)	–	–	–	–
Essential worker, *n* (%)						
Yes	4991 (0.7%)	4958 (0.7%)	117 847 (5.6%)	117 817 (5.5%)	221 514 (8.6%)	221 465 (8.6%)
No	750 064 (99.3%)	750 097 (99.3%)	2 005 481 (94.4%)	2 005 511 (94.5%)	2 366 804 (91.4%)	2 366 853 (91.4%)
Institutionalized, *n* (%)						
Yes	6661 (0.9%)	6589 (0.9%)	17 562 (0.8%)	17 279 (0.8%)	–	–
No	748 394 (99.1%)	748 466 (99.1%)	2 105 766 (99.2%)	2 106 049 (99.2%)	–	–
Socio-economic level, *n* (%)						
Higher	–	–	620 238 (29.2%)	620 062 (29.2%)	376 578 (14.5%)	376 517 (14.5%)
Intermediate	–	–	643 550 (30.3%)	643 516 (30.3%)	570 719 (22.0%)	570 751 (22.1%)
Lower	–	–	859 540 (40.5%)	859 750 (40.5%)	1 641 021 (63.4%)	1 641 050 (63.4%)
Foreign, *n* (%)						
Yes	81 524 (10.8%)	81 495 (10.8%)	–	–	–	–
No	673 531 (89.2%)	673 560 (89.2%)	–	–	–	–
Comorbidities, *n* (%)						
Yes	326 172 (43.2%)	326 163 (43.2%)	549 014 (25.9%)	549 024 (25.9%)	1 109 074 (42.8%)	1 109 051 (42.8%)
No	428 883 (56.8%)	428 892 (56.8%)	1 574 314 (74.1%)	1 574 304 (74.1%)	1 479 244 (57.2%)	1 479 267 (57.2%)
Immune status, *n* (%)						
Yes	22 630 (3.0%)	22 621 (3.0%)	60 697 (2.9%)	60 477 (2.8%)	575 741 (22.2%)	575 687 (22.2%)
No	732 425 (97.0%)	732 434 (97.0%)	2 062 631 (97.1%)	2 062 851 (97.2%)	2 012 577 (77.8%)	2 012 631 (77.8%)

a Each day of the enrolment period (1 January 2021 to 1 September 2021), newly primary vaccinated individuals (*exposed*) were 1:1 matched to non-vaccinated or partially vaccinated individuals (*unexposed*) based on a set of confounders, resulting in the matched study population.

Propensity score distributions before and after matching are visualized in Supplementary Fig. S7, and SMDs are in Supplementary Fig. S8 and Supplementary Tables S4–S6.

### Vaccine effectiveness

During the study period, a SARS-CoV-2 infection was documented in 34.4% (Aragon), 26.6% (Brussels and Wallonia), and 15.8% (Finland) matched individuals. In the exposed group, infection rates were 34.3% (Aragon), 21.9% (Brussels and Wallonia), and 16.0% (Finland), while 34.6%, 31.2%, and 15.7% in the unexposed group, respectively.

**Figure 2.**
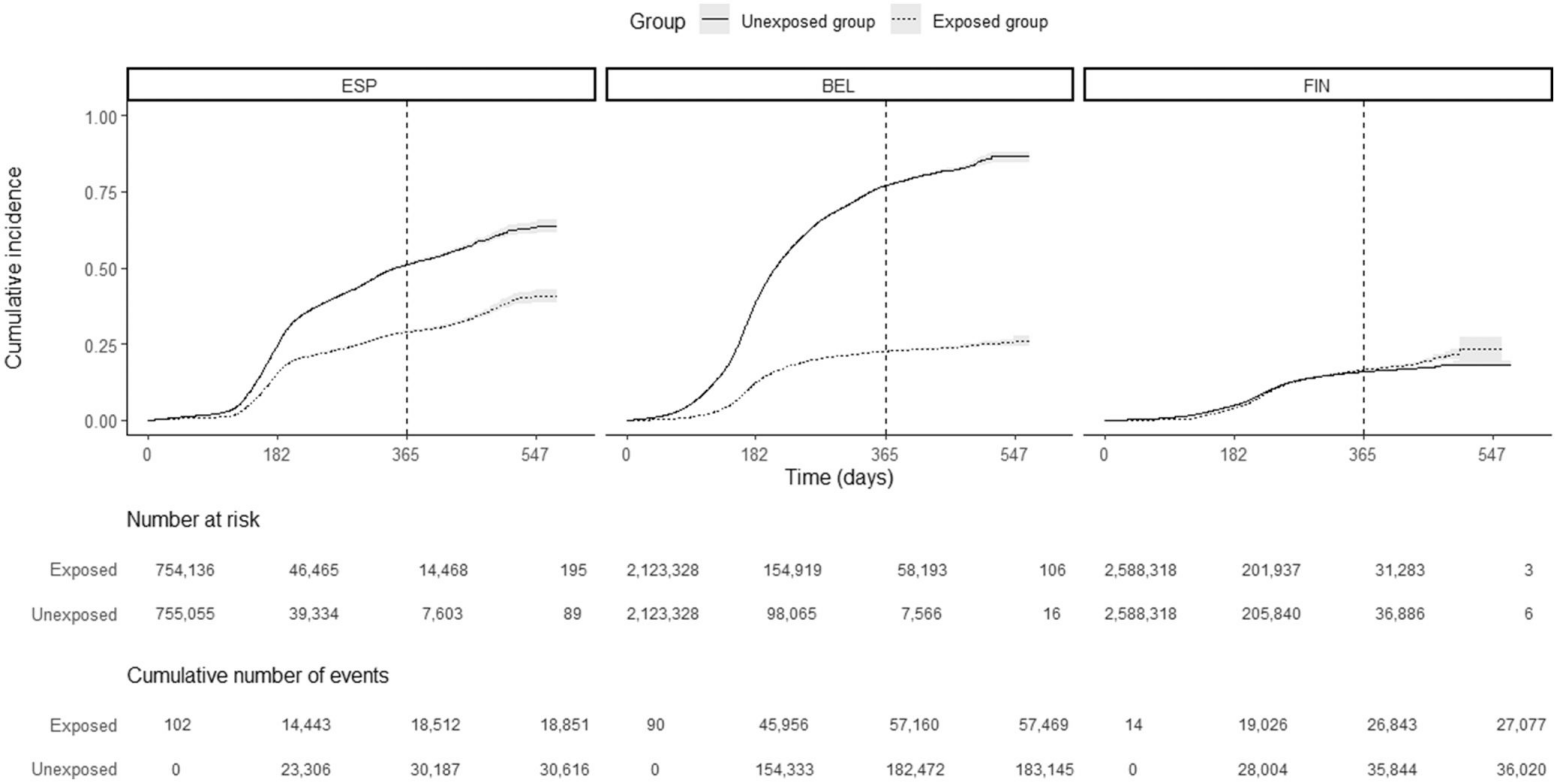
Cumulative incidence (CInc) curves of documented SARS-CoV-2 infections for both exposure groups (unexposed group versus exposed group), obtained from local analysis in the different sites: Aragon, Spain (ESP); Brussels and Wallonia, Belgium (BEL); Finland (FIN). CInc(t) = 1 − *S*(*t*), with *S*(*t*) the survival probability at time *t*. Each day of the enrolment period (1 January 2021 to 1 September 2021), newly primary vaccinated individuals (exposed) were 1:1 matched to un- or partially vaccinated individuals (unexposed) based on a set of confounders, resulting in the matched study population. Study period: 1 January 2021 to 1 September 2022. A vertical dotted line at 365 days indicates the time up to which the restricted mean survival time is estimated.

Cumulative incidence curves diverged in Aragon and Brussels and Wallonia, with more cases in the unexposed group. In Finland, the curves negligibly diverged over 365 days of follow-up (Fig. 2).

Primary vaccination extended the average free-of-infection time by 35.9 days [95% confidence interval (CI) (34.9–37.0), *P* values <.01] in Aragon (Spain), 59.6 days [95% CI (59.3–60.0), *P* values <.01] in Brussels and Wallonia (Belgium), and 1.6 days [95% CI (1.1–2.0), *P* values <.01] in Finland (Table 2).

**Table 2.** Restricted mean survival times (RMSTs) at 365 days in the contrasted exposure groups (unexposed group versus exposed group) and restricted mean survival time difference (RMSTD, RMST exposed group—RMST unexposed group) resulting from local analysis in the different sites: Aragon (Spain), Brussels and Wallonia (Belgium), and Finland^a^.

Measure	Aragon (Spain)	Brussels and Wallonia (Belgium)	Finland
RMST [95% CI], unexposed group (days)	280.758 [279.943–281.573]	214.444 [214.144–214.745]	340.580 [340.268–340.893]
RMST [95% CI], exposed group (days)	316.696 [316.035–317.357]	274.070 [273.862–274.278]	342.145 [341.807–342.483]
RMSTD [95% CI] (days)	35.938 [34.889–36.988]	59.626 [59.260–59.991]	1.565 [1.104–2.025]
*P*-value RMSTD	<.001	<.001	<.001

a Each day of the enrolment period (1 January 2021 to 1 September 2021), newly primary vaccinated individuals (exposed) were 1:1 matched to un- or partially vaccinated individuals (unexposed) based on a set of confounders, resulting in the matched study population. Study period: 1 January 2021 to 1 September 2022.

### Sensitivity analyses

E-values indicate that unmeasured confounders would need to be associated with both exposure and outcome with magnitudes of 2.464 in Aragon (Spain), 4.879 in Brussels and Wallonia (Belgium), and 1.691 in Finland (HR scale) to falsate the observed effects.

In Brussels and Wallonia, the SES-unadjusted RMSTD was compared to the SES-adjusted, with a view to test potential changes in the estimate of the ATE when considering SES as a confounder showing no statistically significant differences [Supplementary Fig. S8: SES-unmatched RMSTD = 57.734 (57.378–58.090) vs. SES-matched RMSTD = 59.626 (59.260–59.991)—Table 2].

## Discussion

Vaccination campaigns were crucial in the COVID-19 pandemic response. The evaluation of the real-life effectiveness of this population-based vaccination program should serve to inform future health policies. This study investigated SARS-CoV-2 primary vaccination effectiveness across Aragon, Brussels/Wallonia, and Finland using real-world data. The primary vaccination programs were effective in prolonging the average time free of infection in Aragon (35.9 days) and Brussels and Wallonia (59.6 days), but showed a minor difference in Finland (1.6 days). Given that this protective period of 36–60 days (in Aragón and Brussels and Wallonia) is several times longer than the COVID-19 infectious period of 10-12 days, it directly and effectively reduces the number of susceptible individuals in the population at any given moment, consequently reducing the attack rate. By temporarily removing a large segment of the population from the at-risk pool, the vaccine’s effectiveness translates into slower, less intense spread of the virus.

Primary vaccination programs effectively prevented SARS-CoV-2 infections in Aragón (Spain), Brussels and Wallonia (Belgium), and Finland, and results from this study were consistent with prior studies (del Cura-Bilbao *et al.* [5] (Aragon), Braeye *et al.* [4] (Belgium), Baum *et al.* [6], and Poukka *et al.* [21] (Finland)) on specific population subgroups and contexts.

### Strengths and limitations of the study

Main strengths in this study included a population approach to the effectiveness of the vaccination programs derived from virtually the whole population of three different nations, using detailed individual-level information from high-quality, routinely collected population health registries. Data linkage between several data sources enabled controlling for relevant confounders. In addition, the iteratively daily propensity score matching, using the information available up until each day of the study, resulted in balanced exposure groups at a population level within each residence area, while also taking into account time-dependent and contextual factors, such as the variants prevalent during the study period in each region or the health policy measures other than the vaccine that were concurrently applied during that time. Finally, we estimated E-value to understand the potential effect of unmeasured confounders. E-values lower than certain confounders’ associations introduced in the survival model [e.g. HR of 2.89 (2.86–2.93) for the factor age, category 5] indicate the plausibility of unmeasured confounding to explain away the effect estimate. The estimated E-values suggest that unmeasured confounders are unlikely to challenge causality in Aragon and Brussels and Wallonia, but unmeasured confounding is plausible in Finland (1.691). The study followed the framework for approaching federated causal inference published elsewhere (Meurisse *et al.* [11]) meant to generate reproducible and comparable results.

On the other hand, the accuracy of these estimates in each population may be influenced by various limitations including data availability. Iteratively daily matching, using propensity-scores based on the information available up to that point, aimed to create comparable exposure groups, achieved good covariate balance required to assure conditional exchangeability (Table 1, and Supplementary Tables S4–S6). However, matching was limited to available confounders, and residual confounding might persist as covariates with high or complete missing were excluded as matching factors in the local analyses, for example, information on individual-level SES was lacking for the population cohort in Aragon (Spain). SES was considered to be associated with both SARS-CoV-2 vaccine uptake and the risk of contracting SARS-CoV-2 based on previous studies [22–25] and, therefore, recognized as a potential confounder in the exposure-outcome relationship. It was hypothesized that adjusting for residence area (NUTS3 level) could achieve comparability in area-level socio-economic differences between the exposure groups. However, within area differences might remain that can only be resolved at a smaller area or individual level and residual confounding could persist. Therefore, additionally, we presume the need to adjust for lower-level socioeconomic differences, approached here by matching on individual-level SES. To assess the potential impact of residual confounding by individual-level SES, the magnitude of confounding by this covariate was estimated for the Brussels and Wallonia population cohort (Belgium). The limited percentage change (3.2%) in the estimate (see Supplementary Table S7) that was obtained does not give a clear indication of confounding by individual-level SES in this analysis, already controlled at area-level; all in all, the extrapolation of this result to the other cohorts would not necessarily apply. Finally, other missing variables that may translate into residual confounding are information on the covariates foreign and pregnancy in Belgium, institutionalized, foreign and pregnancy in Finland. In addition to the potential for residual confounding in Finland, certain groups could be potentially underrepresented (e.g. marginalized communities, those with limited access to healthcare and therefore not reflected in administrative and clinical data sources) which could have also affected the observed results. Another limitation of the study is the lack of accounting for competing risks, particularly the risk of death, in the survival analysis, although both, exposed and non-exposed matched individuals were censored upon one dying, mitigating the potential effect. While this could influence the interpretation of local results, we assume that due to the similar death rates across countries, the impact on our cross-country comparisons were limited. Finally, we need to acknowledge that assumptions on our exposure-outcome relationship as defined in the predefined causal model may not hold over time, and therefore there is still the need to continuously monitor vaccine effectiveness to assess the impact on population health, guide, and adapt policy over time.

On a different note, reliance on SARS-CoV-2 test data, which is influenced by characteristics of the testing program and test-seeking behaviour, could have also introduced bias. Although testing strategies were adjusted multiple times during the study period in the considered sites, testing mainly targeted individuals with a high probability of infection (i.e. symptomatic individuals suspected of having COVID-19) or was performed for screening purposes [26–29]. Our expectation is that this approach, along with the matching strategy, based on a daily basis within the area-of-residence approach, may have mitigated the residual confounding due to different testing strategies between the two exposure groups.

Nonetheless, this strategy does not prevent potential failures in the exclusion criteria (e.g. exclusion of individuals with a previous SARS-CoV-2 infection) in our study, a factor that may be behind the almost negligible effect of the Finnish cohort. Unlike testing strategies in Aragon (Spain) and Brussels and Wallonia (Belgium), Finland focussed on testing individuals with symptoms indicative of COVID-19, overlooking asymptomatic high-risk contacts identified by contact tracing as per their health policy at the time. Seroprevalence studies conducted in the participating regions indicate that reported COVID-19 cases represented only a fraction of the actual circulation (approached through the prevalence of viral antibodies) during 2020 and 2021 [30–33]. As such, excluding individuals with a prior SARS-CoV-2 infection to eliminate the element of natural immunity in our study, solely based on registered PCR tests, might not be sufficient.

In addition, resorting to testing data (i.e. PCR tests) to assess infection may have introduced potential misclassification of the outcome as countries deployed different testing strategies during the different stages of the pandemic and health policy was issued also regarding testing. Kuitunen *et al.* [34] reported higher testing rates among vaccinated individuals, in a retrospective observational study in the Southern Savonia region of Finland, underscoring the potential bias introduced by testing frequency. Bias due to non-exhaustive or non-random testing might be present in all obtained vaccination effectiveness estimates, which advises a cautious interpretation of the vaccination effectiveness estimate obtained for the Finnish population cohort due to this limitation. The results of the study should be interpreted taking into account these limitations and the potential residual biases.

### Comparative assessment

Real-world multisite evidence frequently lacks comparability due to variations in methodological approaches, exposure definitions (e.g. the definition of ‘primary vaccination’, considered vaccination schedules but does not account for the prevalence of the diverse schedules in each region), outcome definitions (e.g. SARS-CoV-2 infections, COVID-19 hospitalization, severe clinical outcomes and how they are registered), and/or target populations (e.g. the entire population of a region or country *versus* individuals with certain characteristics such as nursing home residents). This study used a reproducible approach to obtain internally accurate and methodologically comparable estimates, thus, allowing a more thorough interpretation of the differential effectiveness across sites; for example, whether the other COVID19 policies concurrent to the vaccination program or other contextual factors such as higher virus circulation [30, 32, 33], higher disease prevalence [35], or differences in population density [36–39] could be explaining the difference in effectiveness.

Notably, the sites with the highest observed VE estimates were those with the highest measured population density in 2021: 28.0 inhabitants/km^2^ in Aragon, 3889.0 inhabitants/km^2^ in Brussels and Wallonia, and 18.2 inhabitants/km^2^ in Finland. However, it is essential to recognize that not all individuals within the population undergo testing at any given time. As such, the observed incidence can only serve as a proxy for the real incidence, and testing strategies might importantly impact key indicators. For example, asynchronous COVID-19 waves and variation in the prevailing viral variants, with certain variants being more efficient in evading vaccine-induced immunity, could be explaining part of the difference in the observed effectiveness of vaccines across the three countries. Linking research data from viral gene sequencing collections to real-world data poses challenges due to limited coverage in existing research datasets and their lack of representativeness.

### Implications

Assessing population-wide real-world SARS-CoV-2 vaccination effectiveness provides crucial insights for evidence-driven policies and fortifying our joint efforts in combating COVID-19 and other infectious diseases. It enables continuous monitoring of vaccine performance in real-world scenarios, extending beyond clinical trials, and facilitates the evaluation of public health interventions. By acquiring such evidence, we can shape impactful public health strategies and optimize cross-border vaccination campaigns. Furthermore, acquiring accurate estimates plays a crucial role in effectively communicating vaccine benefits to the public and addressing concerns regarding vaccination.

This study demonstrates a methodological framework for federated causal inference, highlighting the feasibility of conducting cross-country large-scale public health interventions evaluation, while providing a mechanism that preserves all the ethical and legal issues regarding the use of individual personal data paving the way for future research.

## Conclusion

This federated population-based observational studied the effectiveness of the SARS-CoV-2 primary vaccination campaign in Aragon (Spain) and Brussels and Wallonia (Belgium) in preventing SARS-CoV-2 infections. The vaccination showed to be effective in Aragon (Spain) and Brussels and Wallonia (Belgium) although the observed effect was negligible in Finland. Questions remain about the causal nature of the estimated effects as there is potential for residual confounding and some selection bias. The application of this federated methodological approach has demonstrated to be effective in assessing population-based policies that rely on sensitive population, health, and care data.

## Supplementary Material

ckaf247_Supplementary_Data

## Data Availability

The research objects generated during the current study (i.e. a study protocol, a data management plan, a causal model, a common data model, a synthetic dataset, an interoperable analysis pipeline, and local outputs) are available from Zenodo [12, 15, 16, 23] and GitHub, https://w3id.org/ro/doi/10.5281/zenodo.6913045. The individual-level datasets analysed during the current study do not fulfil the requirements for open data access. Data access to registry data from Aragon (through the BIGAN platform) is managed by the Aragon Institute of Health Sciences and requires prior approval of the research protocol by the Clinical Research Ethics Committee of Aragon (CEICA). Data access to registry data from Brussels and Wallonia (through the healthdata.be platform) within the LINKVACC project is granted ad nominatum for the scientists involved in the surveillance activities at Sciensano. External investigators with a request for selected data should fill in the data request form (https://epistat.sciensano.be/datarequest/, accessed on 26 May 2024). Depending on the type of desired data (anonymous or pseudonymized), the provision of data will have to be assessed by the Belgian Information Security Committee Social Security and Health based on legal and ethical regulations and is outlined in a data transfer agreement with the data owner (Sciensano). All Finnish registry data is available through the register authorization office FinData (www.findata.fi).
