# Supplementary material for: Real-world comparative effectiveness of SARS-CoV-2 primary vaccination campaigns against SARS-CoV-2 infections: a federated observational study emulating a target trial in three nations
Source: Eur J Public Health. 2026 Jan 7;36(2):ckaf247. doi: 10.1093/eurpub/ckaf247 (PMC13064525; doi:10.1093/eurpub/ckaf247)
Supplement: ckaf247_Supplementary_Data [file ckaf247_supplementary_data.zip › ejph-2025-05-om-0441-File004.docx]

## Supplementary Materials

**Description:**

This Supplementary provides readers with supplementary information about the study.

**Section A**: Target trial specification

In the target trial, individuals eligible to be vaccinated (aged 5 to 115 - included) in Aragon (Spain), Brussels or Wallonia (Belgium), or Finland, excluding individuals that had experienced a SARS-CoV-2 infection before potential enrolment, would have been eligible for inclusion in the study population. Eligible individuals would have been enrolled in the study from the start of the SARS-CoV-2 vaccination campaign (January 1, 2021) until the beginning of the SARS-CoV-2 booster campaign (1 September 2021), randomly assigning them to one of the exposure arms (a) completing a primary vaccination schedule (defined as two doses of BNT162b2, mRNA-1273, or ChAdOx1-S or one dose of Ad26.COV2.S), *versus* (b) not completing a primary vaccination schedule (either not receiving any vaccine dose or being only partially vaccinated, i.e., one dose of BNT162b2, mRNA-1273, or ChAdOx1-S). Thus, the specified enrolment period covered nine months. Enrolled individuals would have been followed up from enrolment (i.e., at randomisation to one of the vaccination strategies, baseline t_0_) until either (1) the end of the follow-up period, 365 days after baseline, (2) the experience of a diagnosed SARS-CoV-2 infection (i.e., the outcome of interest), (3) death (all causes), (4) loss to follow-up, (5) the date of receiving a booster dose, whichever occurs first. Data could have been collected until September 1, 2022, allowing a potential follow-up of 365 days (12 months) for individuals enrolled (baseline t_0_) at the end of the enrolment period. The timeline of the specified hypothetical target trial is displayed in Figure S1.

**
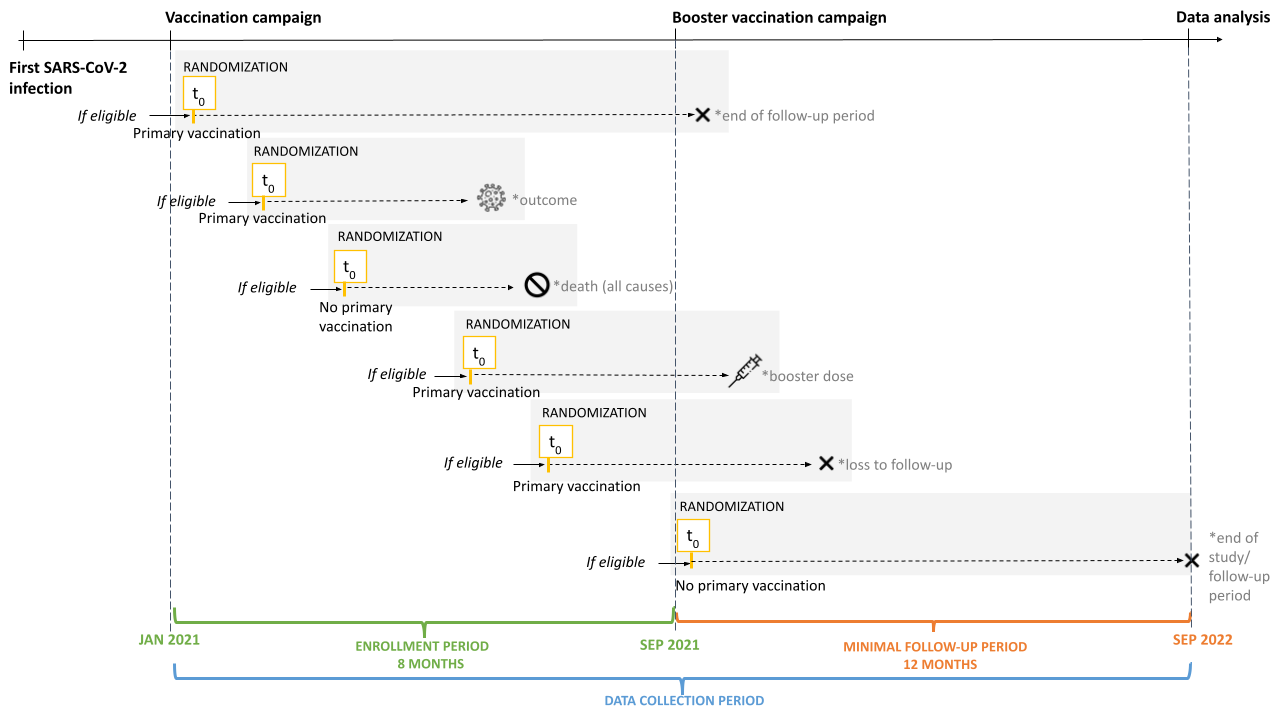
**

**Figure S1.** Timeline of the target trial. t_0_: baseline, at which individuals would have been randomised to one of the intervention arms.


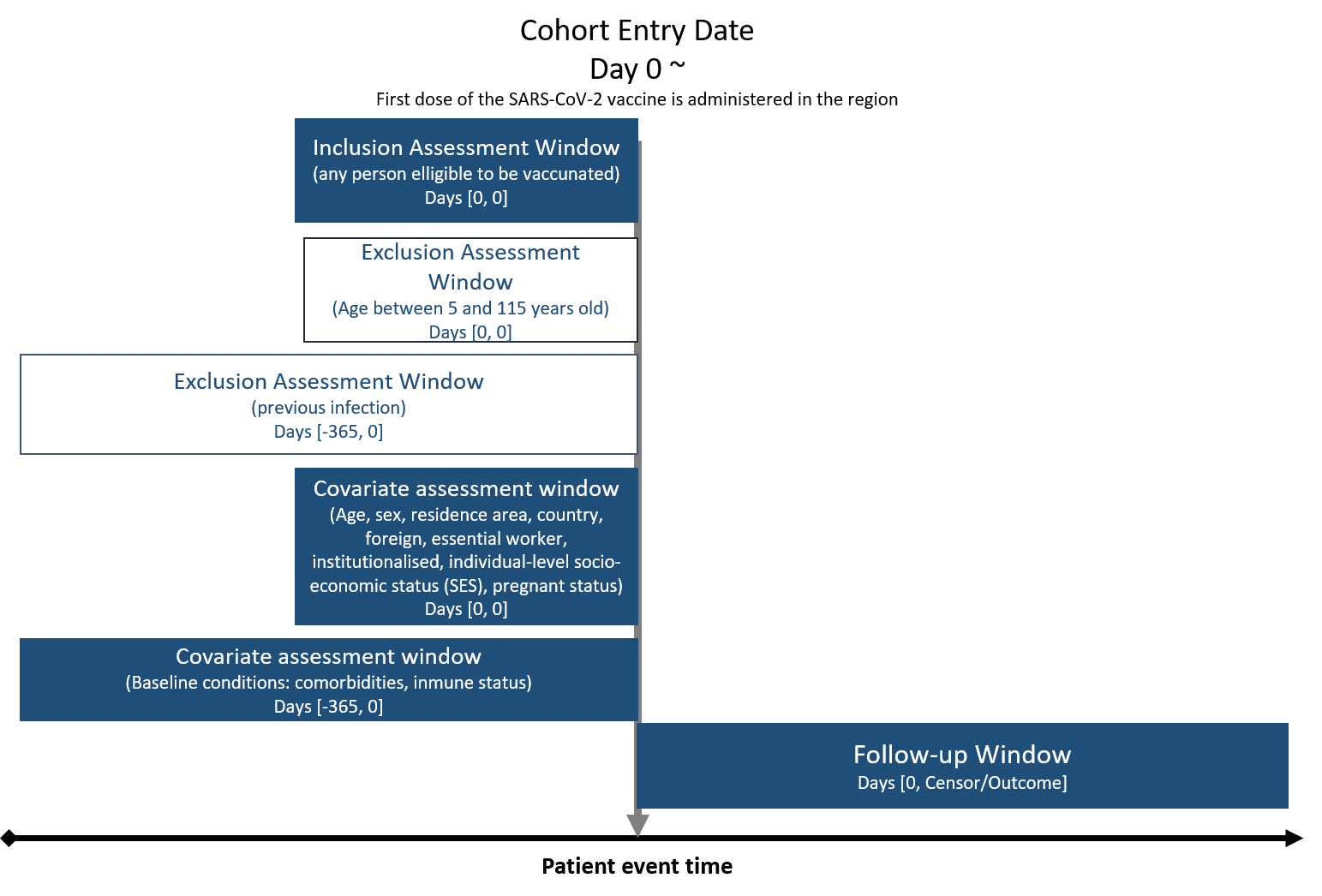


**Figure S2.** Study design diagram accounting for patient event time from the cohort entry date to the end of the follow-up window (i.e., first booster dose administration in the region).

**Section B**: Target trial protocol and emulation using observational data

**Table S1.** Protocol of the target randomised control trial and target trial emulation by using observational data.

| **Protocol Component** | **Target Trial Specification (*Ideal Hypothetical Trial*)** | **Target Trial Emulation (*Actual Study Design reusing Real World Data*)** |
| --- | --- | --- |
| **Eligibility criteria** | - **Study population:** All individuals eligible to be vaccinated. - **Enrolment period**: From the start of the SARS-CoV-2 vaccination campaign (January 1, 2021) to the start of the SARS-CoV-2 booster vaccination campaign (September 1, 2021). - **Age**: 5 to 115 years old, included (*all individuals eligible for vaccination*). - **Previous infection**: Individuals without a SARS-CoV-2 infection before enrolment. - **Country**: Individuals living in the respective country | - **Study population**: All individuals vaccinated with at least one dose of the SARS-CoV-2 vaccine (any of the available brands) and all individuals eligible to be vaccinated with a documented positive diagnosis (irrespective of the type of test) for SARS-CoV-2 infection during the data extraction period. - **Enrolment period**: From the start of the SARS-CoV-2 vaccination campaign (January 1, 2021) until the beginning of the SARS-CoV-2 booster vaccination campaign (September 1, 2021). - **Age**: 5 to 115 years old, included (*all individuals eligible for vaccination*). - **Previous infection**: Individuals without a documented confirmed infection before completing the primary vaccination schedule (i.e. enrolment) or before January 1, 2021 (SARS-CoV-2 vaccine roll-out) for those not having completed a primary vaccination schedule (controls). - **Country**: Resident of the respective country   We applied these eligibility criteria to the data obtained by fully joining the registered individuals in the COVID-19 cases and vaccination datasets. Therefore, individuals with neither a registered vaccine dose nor a positive diagnosis during the data extraction period were excluded. |
| **Treatment strategies** | Primary analysis  1) Exposed group: Completing a primary vaccination schedule (defined as two doses of BNT162b2, mRNA-1273, or ChAdOx1-S or one dose of Ad26.COV2.S)  2) Unexposed group: Not completing a primary vaccination schedule (either not receiving any vaccine dose or being only partially vaccinated, i.e., one dose of BNT162b2, mRNA-1273, or ChAdOx1-S).  Pragmatic trial: strategies compared under the usual conditions. | Same as in Target Trial Specification.  Vaccination status ascertainment (including brand, dose and date) based on the vaccination registry.  The date of being primary vaccinated (completing a primary vaccination schedule) was considered 14 days after receiving the final dose of the primary vaccination schedule. |
| **Assignment procedures** | At baseline, participants will be randomly assigned to either complete the primary vaccination schedule (*exposed* *group*) or not complete it (*unexposed group*). The participants will be aware of the strategy to which they have been assigned (i.e., without blind assignment). | To mimic randomization, we needed to adjust for all confounding factors required to ensure comparability (conditional exchangeability) of the groups defined by vaccination (*as identified in the Directed Acyclic Graph* [[1]](https://www.zotero.org/google-docs/?ccpFWJ)). We assumed that individuals were randomly assigned within levels of baseline covariates: age, gender, residence area, country, foreign, essential worker, institutionalised, individual-level socio-economic status (SES), presence of comorbidities, immune status and pregnancy. A detailed covariate description could be found in the specified Common Data Model (CDM) [[1]](https://www.zotero.org/google-docs/?cSs4M1). The adjustment was performed via matching. |
| **Follow-up period** | - Starts, for each individual, at randomization to one of the vaccination strategies (baseline) - Ends at diagnosis of SARS-CoV-2 infection (i.e., outcome of interest), death, loss to follow-up, the date of receiving a booster dose, end of the follow-up period (365 days after baseline), whichever occurs first. | Sequential trial emulation:   - Starts, for each individual:   - Exposed group: time of completing a primary vaccination schedule   - Unexposed group: first eligible time (time of completing a primary vaccination schedule of the matched pair) - Ends at diagnosis of SARS-CoV-2 infection (i.e., the outcome of interest), death (all causes), death (all causes) of matched individual, completion of a primary vaccination schedule (for not/partially vaccinated individuals*, unexposed group*), completion primary vaccination of the matched control (for primary vaccinated individuals, *exposed group*), administration of a booster dose (for primary vaccinated individuals, *exposed group*), administration of a booster dose of the matched primary vaccinated individual (for not/partially vaccinated individuals, *unexposed group*), the end of the study period (i.e., September 1, 2022, one year after the end of the enrolment period). Loss to follow-up due to emigration or ‘disenrollment’ from the healthcare system was not taken into account.   Newly vaccinated individuals (completing a primary vaccination schedule) were eligible for inclusion in the *exposed group* of the study, even if they had previously been selected in the *unexposed group* (as controls). |
| **Outcome** | Laboratory-confirmed SARS-CoV-2 infection | A documented laboratory-confirmed SARS-CoV-2 infection, excluding self-reported test results. |
| **Causal contrast of interest** | Per-protocol effect. | Observational analogue of the per-protocol effect. |

**Section C:** The constructed causal model using a Directed Acyclic Graph


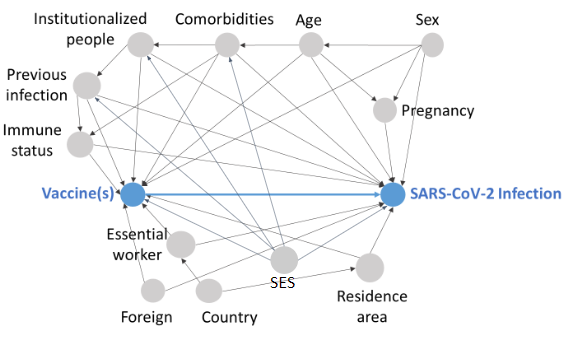

**Figure S3.** The defined causal model, constructed using a Directed Acyclic Graph (DAG), capturing assumptions on variables and their relationships. SES: Socio-economic status, measured at the individual level.

**Section D:** Decision algorithm to guide decisions on how to manage missing values

**
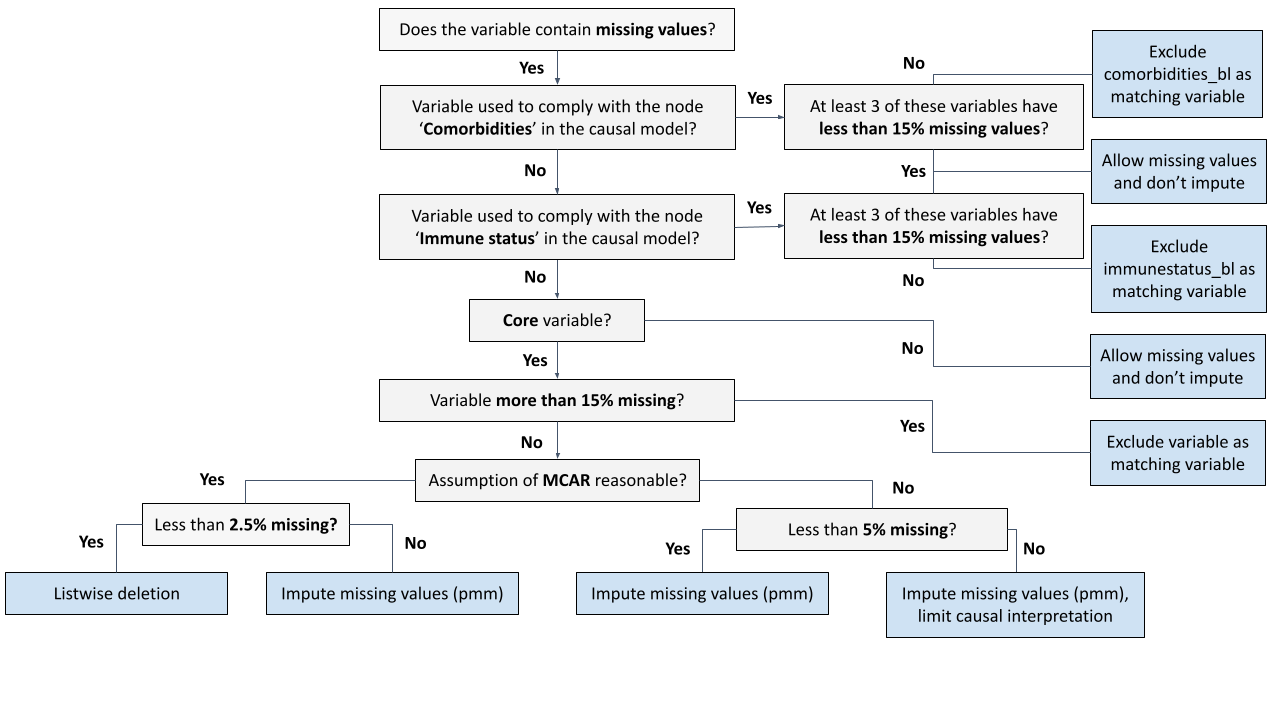
**

**Figure S4.** Decision tree for the managing of missing values for further analytical steps. For each variable, different checks are conducted, based on which a decision is made on handling missing values. Variables used to comply with the node ‘*Comorbidities*’ in the causal model (i.e., used to compute ‘*comorbidities_bl’* in the analytical pipeline): diabetes_bl, obesity_bl, heart_failure_bl, copd_bl, solid_tumor_without_metastasis_bl, chronic_kidney_disease_bl, sickle_cell_disease_bl, hypertension_bl, chronic_liver_disease_bl. Variables used to comply with the node ‘*Immune status*’ in the causal model (i.e., used to compute ‘*immunestatus_bl’* in the analytical pipeline): blood_cancer_bl, transplanted_bl, hiv_infection_bl, primary_ immunodeficiency_bl, immunosuppression_bl. Core variables: age_nm, sex_cd, residence_area_cd, pregnancy_bl, essential_ worker_bl, institutionalized_bl, foreign_bl and socecon_lvl_cd. Missing values in core variables will obstruct subsequent analysis steps. MCAR: missing completely at random; Is it reasonable to assume MCAR in the subset of variables used to predict missing values (core variables plus variables used to comply with the node ‘*Comorbidities*’ and ‘Immune status’ in the causal model, with less than 15% missing values)? The MCAR's reasonableness is tested using Little’s test [[2]](https://www.zotero.org/google-docs/?FaDu0B). Pmm: predictive mean matching (matching method).

**Section E:** Matching algorithm

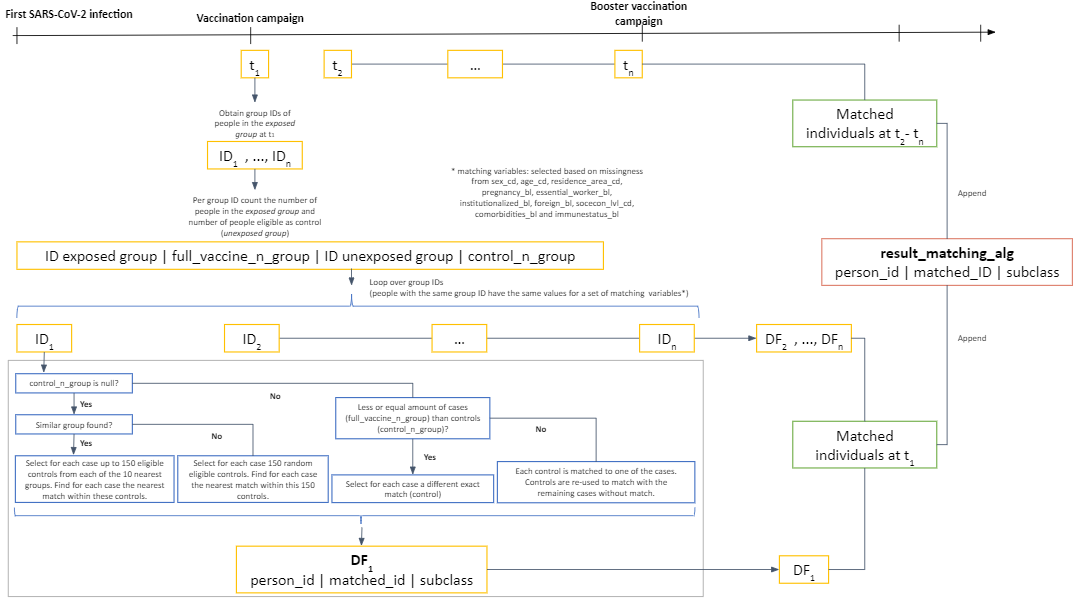


**Figure S5.** Graphical overview of the automated matching process. t1-tn: days considered within the enrolment period. Group ID: ID of groups identified based on combinations of considered covariates (i.e., matching variables). People in the *exposed group* at tx: individuals completing their primary vaccination schedule at the day tx. ‘result_matching_alg’: DuckDB database table, used to store matching results.

**Section F:** Timeline of the sequentially emulated target trial


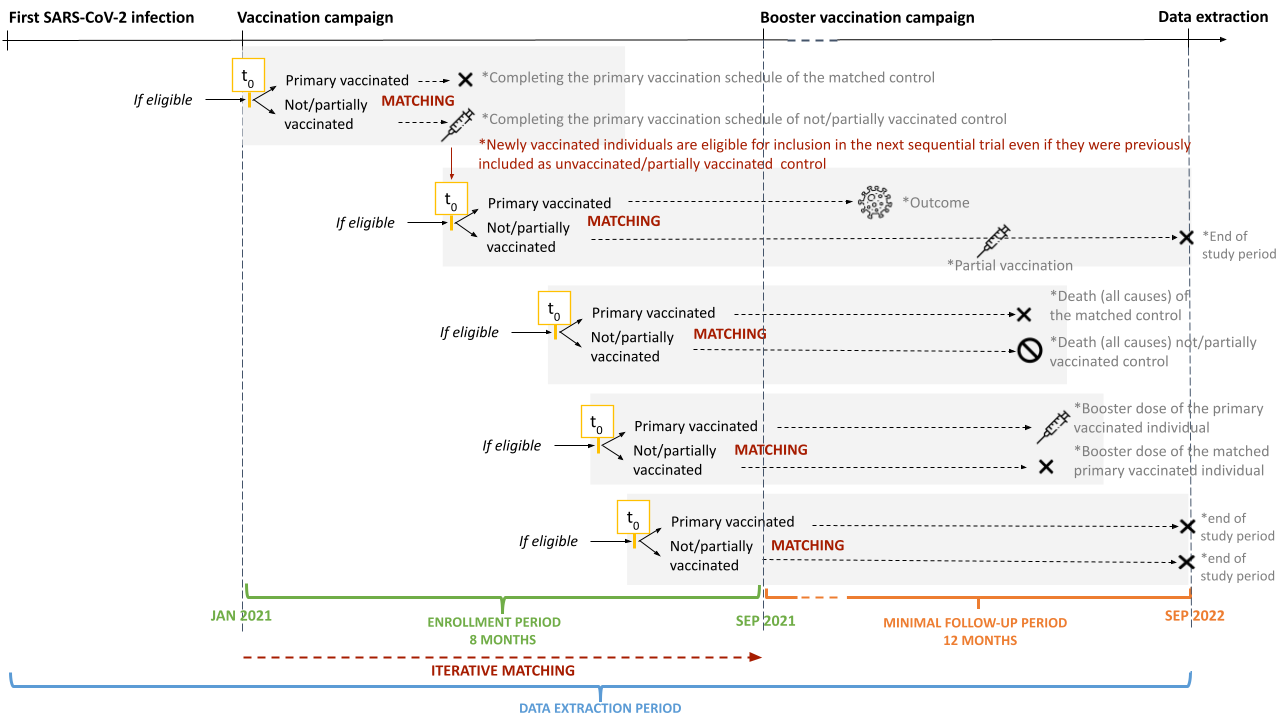


**Figure S6.** Timeline of the sequentially emulated target trial by reusing observational data within each participating region or country. t_0_: baseline, at which the vaccination status of individuals is assessed, and matching is performed. Enrolment period: January 1, 2021, to September 1, 2021. Data extraction period: from the date of the first documented SARS-CoV-2 infection until the most recent date at which data is available at the time of analysis. End of the study period: end of the data extraction period.

**Section G:** Data extraction periods

**Table S2.** Data extraction periods (i.e., starting from the date of the first documented SARS-CoV-2 infection until the most recent date at which data is available at the time of analysis) in the different sites: Aragon (Spain), Brussels and Wallonia (Belgium), and Finland

| **Site** | **Start data extraction period** | **End data extraction period** |
| --- | --- | --- |
| **Aragon (Spain)** | 2020-03-01 | 2023-04-18 |
| **Brussels and Wallonia (Belgium)** | 2020-09-01 | 2024-04-05 |
| **Finland** | 2020-01-01 | 2022-12-20 |

**Section H:** Characteristics of the unmatched population eligible for matching

**Table S3.** Characteristics of the unmatched population eligible for matching by Participant Node (Aragon (Spain), Brussels and Wallonia (Belgium), and Finland) and by intervention group.

|  | **Aragon (Spain)** | | **Brussels and Wallonia (Belgium)** | | **Finland** | |
| --- | --- | --- | --- | --- | --- | --- |
|  | **Primary vaccinated* (N =  755,055)** | **Not primary vaccination ** (N =  244,365)** | **Primary vaccinated*  (N =  2,123,328)** | **Not primary vaccination **  (N = 697,285)** | **Primary vaccinated*  (N =  2,588,318)** | **Not primary vaccination ** (N = 2,263,277)** |
| Sex, n (%) |  |  |  |  |  |  |
| 1 (male) | 360,616 (47.8%) | 126,054 (51.6%) | 1,019,290 (48.0%) | 343,475 (49.3%) | 1,209,685 (46.7%) | 1,184,459 (52.3%) |
| 2 (female) | 394,439 (52.2%) | 118,311 (48.4%) | 1,104,038 (52.0%) | 353,810 (50.7%) | 1,378,633 (53.3%) | 1,078,818 (47.7%) |
| Age (category),  n (%) |  |  |  |  |  |  |
| 2 (5-9 yo) | 0  (0.0%) | 37,945 (15.5%) | 11  (0.0%) | 77,059 (11.1%) | 0  (0%) | 273,060 (12.1%) |
| 3 (10-14 yo) | 318 (0.0%) | 52,280 (21.4%) | 10,611 (0.5%) | 121,717 (17.5%) | 2,242 (0.1%) | 274,985 (12.1%) |
| 4 (15-19 yo) | 16,314 (2.2%) | 33,824 (13.8%) | 80,797 (3.8%) | 86,070 (12.3%) | 37,872 (1.5%) | 224,984 (9.9%) |
| 5 (20-24 yo) | 20,863 (2.8%) | 24,530 (10.0%) | 118,337 (5.6%) | 53,752 (7.7%) | 55,185 (2.1%) | 217,002 (9.6%) |
| 6 (25-29 yo) | 24,610 (3.3%) | 23,541 (9.6%) | 119,579 (5.6%) | 53,672 (7.7%) | 79,327 (3.1%) | 233,172 (10.3%) |
| 7 (30-34 yo) | 36,643 (4.9%) | 16,814 (6.9%) | 134,279 (6.3%) | 56,726 (8.1%) | 99,915 (3.9%) | 210,038 (9.3%) |
| 8 (35-39 yo) | 50,711 (6.7%) | 13,937 (5.7%) | 140,687 (6.6%) | 48,514 (7.0%) | 140,313 (5.4%) | 180,610 (8.0%) |
| 9 (40-44 yo) | 72,485 (9.6%) | 10,592 (4.3%) | 152,447 (7.2%) | 40,357 (5.8%) | 184,719 (7.1%) | 126,999 (5.6%) |
| 10 (45-49 yo) | 79,881 (10.6%) | 8,180 (3.3%) | 170,927 (8.1%) | 32,306 (4.6%) | 195,696 (7.6%) | 90,088 (4.0%) |
| 11 (50-54 yo) | 76,979 (10.2%) | 5,995 (2.5%) | 185,092 (8.7%) | 27,729 (4.0%) | 246,049 (9.5%) | 80,071 (3.5%) |
| 12 (55-59 yo) | 74,495 (9.9%) | 4,556 (1.9%) | 195,044 (9.2%) | 23,020 (3.3%) | 270,896 (10.5%) | 72,732 (3.2%) |
| 13 (60-64 yo) | 66,025 (8.7%) | 4,653 (1.9%) | 194,349 (9.2%) | 18,367 (2.6%) | 280,746 (10.8%) | 61,802 (2.7%) |
| 14 (65-69 yo) | 56,885 (7.5%) | 2,458 (1.0%) | 179,502 (8.5%) | 14,377 (2.1%) | 296,567 (11.5%) | 51,760 (2.3%) |
| 15 (70-74 yo) | 53,710 (7.1%) | 1,276 (0.5%) | 158,875 (7.5%) | 12,431 (1.8%) | 300,864 (11.6%) | 43,919 (1.9%) |
| 16 (75-79 yo) | 45,802 (6.1%) | 1,013 (0.4%) | 110,856 (5.2%) | 9,621 (1.4%) | 179,299 (6.9%) | 30,545 (1.3%) |
| 17 (80-84 yo) | 33,447 (4.4%) | 888 (0.4%) | 76,632 (3.6%) | 7,730 (1.1%) | 125,315 (4.8%) | 34,565 (1.5%) |
| 18 (>=85 yo) | 45,887 (6.1%) | 1,883 (0.8%) | 81,108 (3.8%) | 9,649 (1.4%) | 93,313 (3.6%) | 56,945 (2.5%) |
| Pregnancy, n (%) |  |  |  |  |  |  |
| Yes | 13,395 (1.8%) | 9,341 (3.8%) | - | - | - | - |
| No | 741,660 (98.2%) | 235,024 (96.2%) | - | - | - | - |
| Essential worker, n (%) |  |  |  |  |  |  |
| Yes | 4,991 (0.7%) | 621 (0.3%) | 117,847 (5.6%) | 21,567 (3.1%) | 221,391 (8.6%) | 172,063 (7.6%) |
| No | 750,064 (99.3%) | 243,744 (99.7%) | 2,005,481 (94.4%) | 675,718 (96.9%) | 2,365,934 (91.4%) | 2,048,942 (90.5%) |
| Institutionalised, n (%) |  |  |  |  |  |  |
| Yes | 6,661 (0.9%) | 557 (0.2%) | 17,562 (0.8%) | 1,498 (0.2%) | - | - |
| No | 748,394 (99.1%) | 243,808 (99.8%) | 2,105,766 (99.2%) | 695,787 (99.8%) | - | - |
| Socio-economic level, n (%) |  |  |  |  |  |  |
| Higher | - | - | 620,180 (29.2%) | 133,678 (19.2%) | 376,469 (14.5%) | 311,272 (13.8%) |
| Intermediate | - | - | 643,492 (30.3%) | 187,060 (26.8%) | 570,270 (22.0%) | 633,469 (28.0%) |
| Lower | - | - | 859,436 (40.5%) | 376,455 (54.0%) | 1,640,586 (63.4%) | 1,276,264 (56.4%) |
| Foreign, n (%) |  |  |  |  |  |  |
| Yes | 81,524 (10.8%) | 5,225 (22.2%) | - | - | - | - |
| No | 673,531 (89.2%) | 190,140 (77.8%) | - | - | - | - |
| Comorbidities,  n (%) |  |  |  |  |  |  |
| Yes | 326,172 (43.2%) | 28,768 (11.8%) | 549,014 (25.9%) | 65,621 (9.4%) | 1,109,074 (42.8%) | 430,287 (19.0%) |
| No | 428,883 (56.8%) | 215,597 (88.2%) | 1,574,314 (74.1%) | 631,664 (90.6%) | 1,479,244 (57.2%) | 1,832,990 (81.0%) |
| Immune status, n (%) |  |  |  |  |  |  |
| Yes | 22,630 (3.0%) | 2,016 (0.8%) | 60,697 (2.9%) | 8,303 (1.2%) | 575,741 (22.2%) | 194,678 (8.6%) |
| No | 732,425 (97.0%) | 242,349 (99.2%) | 2,062,631 (97.1%) | 688,982 (98.8%) | 2,012,577 (77.8%) | 2,068,599 (91.4%) |

* Eligible individuals who completed a primary vaccination schedule during the enrolment period (i.e., January 1, 2021, to September 1, 2021)

** Eligible individuals who did not complete a primary vaccination schedule during the enrolment period (i.e., January 1, 2021, to September 1, 2021)

**Section I:** Propensity score distribution in contrasted exposure groups before and after matching in the considered sites

**
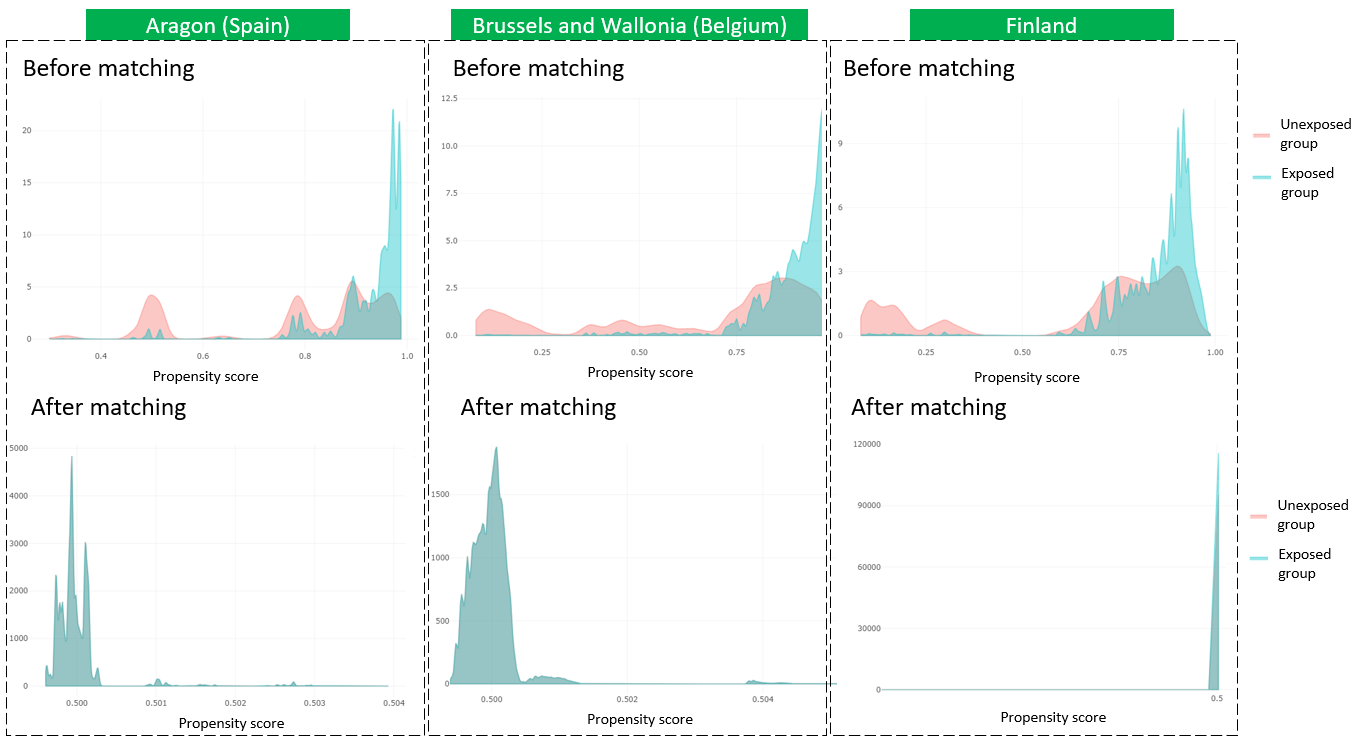
**

**Figure S7.** Propensity score distribution plots in contrasted exposure groups before and after matching in the considered sites: Aragon (Spain), Brussels and Wallonia (Belgium), Finland.

**Section J:** Graphical representation of Standardised Mean Differences (SMDs) before and after matching between contrasted exposure groups for each covariate considered for matching in the different sites

**
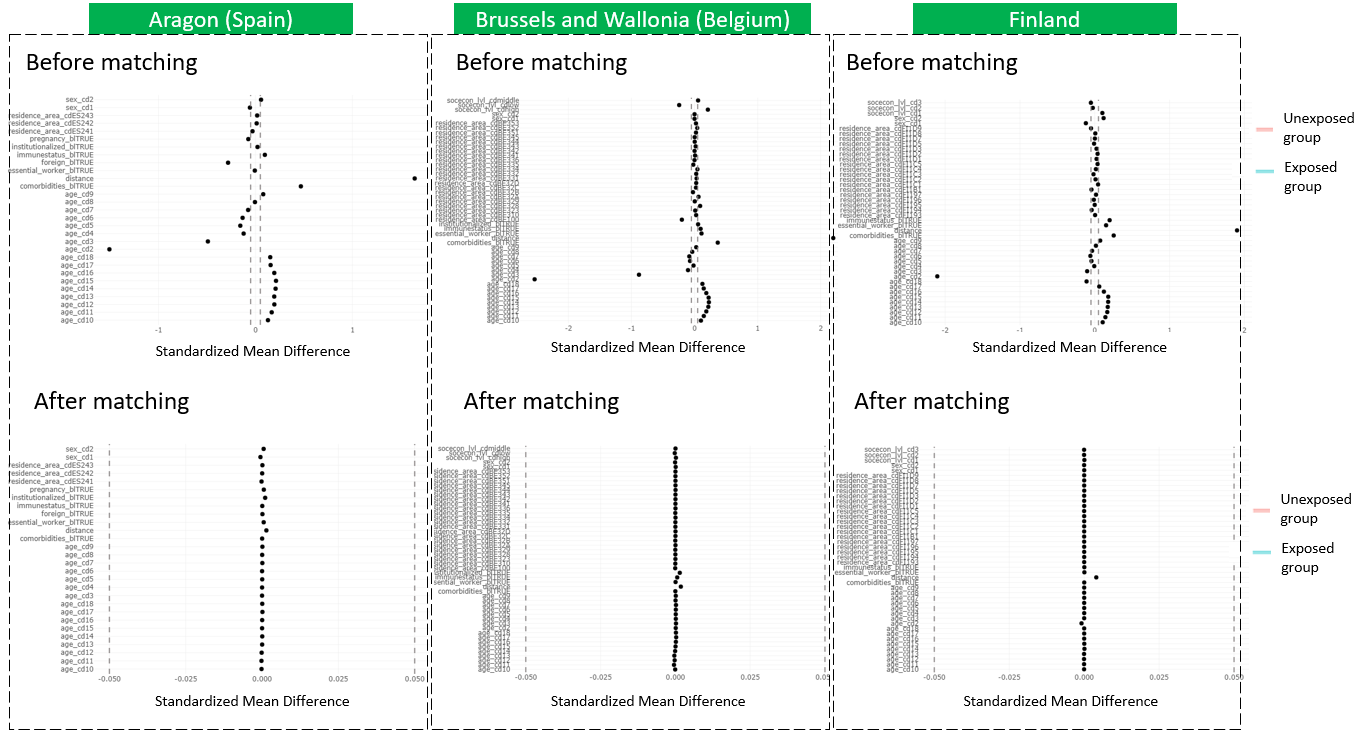
**

**Figure S8.** Graphical representation of Standardised Mean Differences (SMDs) before and after matching between contrasted exposure groups for each covariate considered for matching in the different sites: Aragon (Spain), Brussels and Wallonia (Belgium), Finland.

**Section K:** Standardised Mean Differences (SMDs) before and after matching between contrasted exposure groups for each covariate considered for matching in the different sites

**Table S4.** Standardised Mean Differences before and after matching between contrasted exposure groups for each covariate considered for matching, Aragon (Spain).

|  | **Standardised Mean Difference before matching** | **Standardised Mean Difference after matching** |
| --- | --- | --- |
| Distance | 1.641725020 | 1.412596e-03 |
| sex_cd1 | -0.057432835 | -5.223406e-04 |
| sex_cd2 | 0.057432835 | 5.223406e-04 |
| age_cd2 | -1.506233112 | / |
| age_cd3 | -0.490890385 | 0.000000e+00 |
| age_cd4 | -0.122138493 | 9.109062e-06 |
| age_cd5 | -0.156618157 | 4.847949e-05 |
| age_cd6 | -0.132791923 | 7.458482e-06 |
| age_cd7 | -0.075861162 | 9.245043e-05 |
| age_cd8 | -0.005404281 | 4.232983e-05 |
| age_cd9 | 0.079957452 | 1.078980e-04 |
| age_cd10 | 0.128666317 | -1.679326e-04 |
| age_cd11 | 0.167351509 | -1.882102e-04 |
| age_cd12 | 0.194435150 | -1.332369e-04 |
| age_cd13 | 0.193346383 | 2.813053e-05 |
| age_cd14 | 0.207429394 | 6.021460e-05 |
| age_cd15 | 0.211616943 | 1.030473e-05 |
| age_cd16 | 0.193917382 | 3.883785e-05 |
| age_cd17 | 0.156043095 | 1.222994e-04 |
| age_cd18 | 0.152091086 | 6.097802e-05 |
| residence_area_cdES241 | -0.030511385 | -1.641985e-04 |
| residence_area_cdES242 | 0.012192087 | 3.559674e-05 |
| residence_area_cdES243 | 0.017448286 | 1.143012e-04 |
| pregnancy_blTRUE | -0.073902226 | 5.819078e-04 |
| essential_worker_blTRUE | -0.006835831 | 5.393504e-04 |
| institutionalized_blTRUE | 0.021122908 | 1.019759e-03 |
| foreign_blTRUE | -0.282783266 | 1.237589e-04 |
| comorbidities_blTRUE | 0.468086506 | 2.406300e-05 |
| immunestatus_blTRUE | 0.096718282 | 6.990668e-05 |

**Table S5.** Standardised Mean Differences between contrasted intervention groups for each covariate considered for matching, Brussels and Wallonia (Belgium).

|  | **Standardised Mean Difference before matching** | **Standardised Mean Difference after matching** |
| --- | --- | --- |
| distance | 2.1992597402 | 1.828475e-03 |
| sex_cd1 | -0.0001550932 | 9.615221e-05 |
| sex_cd2 | 0.0001550932 | -9.615221e-05 |
| age_cd2 | -2.5354036348 | 1.160008e-04 |
| age_cd3 | -0.8810198701 | 2.040529e-04 |
| age_cd4 | -0.1037882622 | 1.474956e-04 |
| age_cd5 | -0.0160622132 | 1.271869e-04 |
| age_cd6 | -0.0720882998 | 1.524582e-04 |
| age_cd7 | -0.0831480991 | 2.004161e-04 |
| age_cd8 | -0.0368123851 | 5.293941e-05 |
| age_cd9 | 0.0267192980 | 1.363965e-04 |
| age_cd10 | 0.1012464192 | -7.068662e-05 |
| age_cd11 | 0.1466775278 | -4.552732e-04 |
| age_cd12 | 0.1865733535 | -2.327264e-04 |
| age_cd13 | 0.2181298788 | -3.532912e-04 |
| age_cd14 | 0.2274606522 | -8.257133e-05 |
| age_cd15 | 0.2231181058 | 4.819998e-05 |
| age_cd16 | 0.1865803011 | 1.647955e-04 |
| age_cd17 | 0.1458589159 | 2.696093e-04 |
| age_cd18 | 0.1249091665 | 1.865252e-04 |
| residence_area_cdBE100 | -0.2009285148 | -5.906023e-05 |
| residence_area_cdBE310 | 0.0252622841 | -2.544553e-05 |
| residence_area_cdBE323 | 0.0106126766 | 8.193615e-06 |
| residence_area_cdBE328 | 0.0885347426 | -4.255294e-06 |
| residence_area_cdBE329 | 0.0038294871 | -2.408440e-05 |
| residence_area_cdBE32A | 0.0635141327 | 3.229259e-05 |
| residence_area_cdBE32B | -0.0251878781 | -3.402086e-05 |
| residence_area_cdBE32C | 0.0227596552 | 2.658916e-05 |
| residence_area_cdBE32D | 0.0263528162 | 3.787469e-05 |
| residence_area_cdBE331 | 0.0325206280 | 0.000000e+00 |
| residence_area_cdBE332 | 0.0262957873 | -5.503277e-06 |
| residence_area_cdBE334 | 0.0458176653 | 3.477750e-05 |
| residence_area_cdBE335 | -0.0194912089 | -2.916835e-05 |
| residence_area_cdBE336 | -0.0002299111 | 2.795237e-05 |
| residence_area_cdBE341 | 0.0084663934 | 1.044831e-05 |
| residence_area_cdBE342 | 0.0007837180 | 2.460222e-05 |
| residence_area_cdBE343 | 0.0173921956 | 2.009669e-05 |
| residence_area_cdBE344 | 0.0032082133 | 5.086846e-05 |
| residence_area_cdBE345 | 0.0023742138 | 3.290944e-05 |
| residence_area_cdBE351 | 0.0229490948 | 2.887334e-05 |
| residence_area_cdBE352 | 0.0426394233 | 1.622087e-05 |
| residence_area_cdBE353 | 0.0227093472 | 2.235767e-05 |
| essential_worker_blTRUE | 0.1102706999 | 6.170960e-05 |
| institutionalized_blTRUE | 0.0563316624 | 1.471616e-03 |
| socecon_lvl_cdhigh | 0.2112528812 | 1.822808e-04 |
| socecon_lvl_cdlow | -0.2438007833 | -2.014880e-04 |
| socecon_lvl_cdmiddle | 0.0559786835 | 3.484091e-05 |
| comorbidities_blTRUE | 0.3676178677 | -1.075629e-05 |
| immunestatus_blTRUE | 0.0963132700 | 6.217679e-04 |

**Table S6.** Standardised Mean Differences between contrasted intervention groups for each covariate considered for matching, Finland.

|  | **Standardised Mean Difference before matching** | **Standardised Mean Difference after matching** |
| --- | --- | --- |
| Distance | 1.9020165178 | 4.034454e-03 |
| sex_cd1 | -0.1185661440 | 1.703579e-05 |
| ex_cd2 | 0.1185661440 | -1.703579e-05 |
| age_cd2 | -2.1044574284 | -8.790352e-04 |
| age_cd3 | -0.1015375309 | 0.000000e+00 |
| age_cd4 | -0.0066189695 | 9.652817e-06 |
| age_cd5 | -0.0437475031 | 1.069844e-05 |
| age_cd6 | -0.0581121245 | -2.241506e-06 |
| age_cd7 | -0.0358672901 | 1.002754e-05 |
| age_cd8 | 0.0158830199 | -1.194381e-05 |
| age_cd9 | 0.0730296968 | 3.001534e-06 |
| age_cd10 | 0.1061768421 | -2.922819e-05 |
| age_cd11 | 0.1424234383 | -3.424871e-05 |
| age_cd12 | 0.1674116536 | -4.291163e-05 |
| age_cd13 | 0.1736996983 | -3.851477e-05 |
| age_cd14 | 0.1792646857 | 9.339973e-05 |
| age_cd15 | 0.1803915762 | 3.495720e-05 |
| age_cd16 | 0.1224049084 | 0.000000e+00 |
| age_cd17 | 0.0600141517 | 0.000000e+00 |
| age_cd18 | -0.1093198767 | 0.000000e+00 |
| residence_area_cdFI193 | 0.0046732194 | 3.624173e-06 |
| residence_area_cdFI194 | -0.0408667432 | 8.582977e-06 |
| residence_area_cdFI195 | -0.0004519568 | 6.563667e-06 |
| residence_area_cdFI196 | -0.0117511613 | 0.000000e+00 |
| residence_area_cdFI197 | 0.0172933014 | -3.988977e-06 |
| residence_area_cdFI1B1 | -0.0436668134 | -9.252919e-06 |
| residence_area_cdFI1C1 | 0.0443360061 | -2.721600e-06 |
| residence_area_cdFI1C2 | 0.0106594329 | -2.177293e-06 |
| residence_area_cdFI1C3 | -0.0164409409 | 1.938849e-06 |
| residence_area_cdFI1C4 | 0.0155803032 | 0.000000e+00 |
| residence_area_cdFI1C5 | 0.0377920931 | -1.233891e-05 |
| residence_area_cdFI1D1 | 0.0245363444 | 9.299805e-06 |
| residence_area_cdFI1D2 | 0.0380839970 | 0.000000e+00 |
| residence_area_cdFI1D3 | 0.0238659883 | -2.253008e-06 |
| residence_area_cdFI1D5 | -0.0090660344 | 2.024242e-05 |
| residence_area_cdFI1D7 | 0.0020039128 | 0.000000e+00 |
| residence_area_cdFI1D8 | 0.0072482953 | 6.790062e-06 |
| residence_area_cdFI1D9 | -0.0493560583 | 1.501142e-06 |
| essential_worker_blTRUE | 0.1516587655 | 6.767283e-05 |
| socecon_lvl_cd1 | 0.1019365723 | 6.683983e-05 |
| socecon_lvl_cd2 | -0.0259588197 | -2.982094e-05 |
| socecon_lvl_cd3 | -0.0512514925 | -2.325936e-05 |
| comorbidities_blTRUE | 0.2535122393 | 1.795675e-05 |
| immunestatus_blTRUE | 0.1991196427 | 5.016536e-05 |

**Section L:** Change-in-estimate analysis to estimate the magnitude of confounding by individual-level SES

**Table S7.** Restricted Mean Survival Times (RMSTs) in contrasted exposure groups and Restricted Mean Survival Time Difference (RMSTD, RMST intervention group - RMST control group) resulting from local analysis in Brussels and Wallonia (Belgium), with the time-to-event analysis executed on a matched study population obtained by matching on all previously included factors, excluding individual-level SES.

| **Measure** | **Brussels and Wallonia (Belgium)** |
| --- | --- |
| RMST [95% CI], unexposed group | 216.120  [215.828; 216.412] |
| RMST [95% CI], exposed group | 273.854  [273.651; 274.058] |
| RMSTD [95% CI] | 57.734  [57.378; 58.090] |
| P-value RMSTD | <0.001 |

**Reference list**

1. [Estupiñán-Romero F, Van Goethem N, Meurisse M, González-Galindo J, Bernal-Delgado E. BY-COVID - WP5 - Baseline Use Case: SARS-CoV-2 vaccine effectiveness assessment - Common Data Model Specification. 2023. https://doi.org/10.5281/zenodo.7572373.](https://www.zotero.org/google-docs/?g7wwQZ)
2. [Li C. Little’s Test of Missing Completely at Random. The Stata Journal. 2013;13:795–809.](https://www.zotero.org/google-docs/?g7wwQZ)
3. Buuren S van, Groothuis-Oudshoorn K. mice: Multivariate Imputation by Chained Equations. *J. Stat. Softw*. 2021. https://doi.org/10.18637/jss.v045.i03.
4. Ho D, Imai K, King G *et al*. MatchIt: Nonparametric Preprocessing for Parametric Causal Inference. *J. Stat. Softw*. 2011. https://doi.org/10.18637/jss.v042.i08
5. Uno H, Tian L, Horiguchi M *et al*. survRM2: Comparing Restricted Mean Survival Time. 2022. <https://doi.org/10.32614/CRAN.package.survRM2>
6. Mathur MB, Smith LH, Ding P *et al*. EValue: Sensitivity Analyses for Unmeasured Confounding and Other Biases in Observational Studies and Meta-Analyses. 2021.
